# RGB-Stack Light Emitting Diode Modules with Transparent Glass Circuit Board and Oil Encapsulation

**DOI:** 10.3390/ma11030365

**Published:** 2018-03-01

**Authors:** Ying-Chang Li, Yuan-Hsiao Chang, Preetpal Singh, Liann-Be Chang, Der-Hwa Yeh, Ting-Yu Chao, Si-Yun Jian, Yu-Chi Li, Cher Ming Tan, Chao-Sung Lai, Lee Chow, Shang-Ping Ying

**Affiliations:** 1 Green Technology Research Center, Chang Gung University, Kweishan, Taoyuan 333, Taiwan; davenlee15@gmail.com; 2 Department of Electronic Engineering, Chang Gung University, Kweishan, Taoyuan 333, Taiwan; butterhandkimo@hotmail.com (Y.-H.C.); preetpalsingh96@gmail.com (P.S.); masteryeh@yahoo.com.tw (D.-H.Y.); a5884432@yahoo.com.tw (T.-Y.C.); a59609148@yahoo.com.tw (S.-Y.J.); war820331@gmail.com (Y.-C.L.); cmtan@mail.cgu.edu.tw (C.-M.T.); cslai@mail.cgu.edu.tw (C.-S.L.); 3 Department of Otolaryngology, Head and Neck Surgery, Chang Gung Memorial Hospital, Kweishan, Taoyuan 333, Taiwan; 4 Department of Materials Engineering, Ming Chi University of Technology, Taishan, New Taipei City 243, Taiwan; 5 Department of Urology, Chang Gung Memorial Hospital, Kweishan, Taoyuan 333, Taiwan; 6 Department of Nephrology, Chang Gung Memorial Hospital, Kweishan, Taoyuan 333, Taiwan; 7 Department of Physics, University of Central Florida, Orlando, FL 32816, USA; Lee.Chow@ucf.edu; 8 Department of Opto-Electronic System Engineering, Minghsin University of Science and Technology, Xinfeng Hsinchu 30401, Taiwan; sbying@must.edu.tw

**Keywords:** FCLED, cooling oil encapsulation, stacking module, transparent glass circuit board

## Abstract

The light emitting diode (LED) is widely used in modern solid-state lighting applications, and its output efficiency is closely related to the submounts’ material properties. Most submounts used today, such as low-power printed circuit boards (PCBs) or high-power metal core printed circuit boards (MCPCBs), are not transparent and seriously decrease the output light extraction. To meet the requirements of high light output and better color mixing, a three-dimensional (3-D) stacked flip-chip (FC) LED module is proposed and demonstrated. To realize light penetration and mixing, the mentioned 3-D vertically stacking RGB LEDs use transparent glass as FC package submounts called glass circuit boards (GCB). Light emitted from each GCB stacked LEDs passes through each other and thus exhibits good output efficiency and homogeneous light-mixing characteristics. In this work, the parasitic problem of heat accumulation, which caused by the poor thermal conductivity of GCB and leads to a serious decrease in output efficiency, is solved by a proposed transparent cooling oil encapsulation (OCP) method.

## 1. Introduction

Thanks to new materials, new manufacturing processes, and new device configurations, the efficiency of light-emitting diode (LED) products has continuously improved since their wide application in the field of commercial and household illumination. Due to the large demand for white lighting luminescence, the trend is expected to continue for several years [1]. The efficiency of LED products in basic terms is the ratio of light output to the space outside the device and power input to the device, in other words, emitted flux (lumens) divided by power draw (watts). However, there are still several important issues, such as LED packages, thermal handling properties, and optical loss, which affect the total quality of LED devices [2,3,4,5,6,7]. Of course, LED package efficiency also has many variables, such as the method of generating white light, color quality, and drive methodology. At the present time, there are two primary methods for generating white light with LEDs: phosphor conversion (PC) and RGB color mixing. Currently, PC-LEDs are the most adopted commercial option. However, due to additional inefficiencies related to phosphor conversion, PC-LED packages are thought to have lower theoretical efficiency than that of RGB mixed systems. There is another reason for the consideration of RGB LED—its color-tunable ability—which provides additional options for color lighting applications [8,9,10,11].

**Figure 1:**
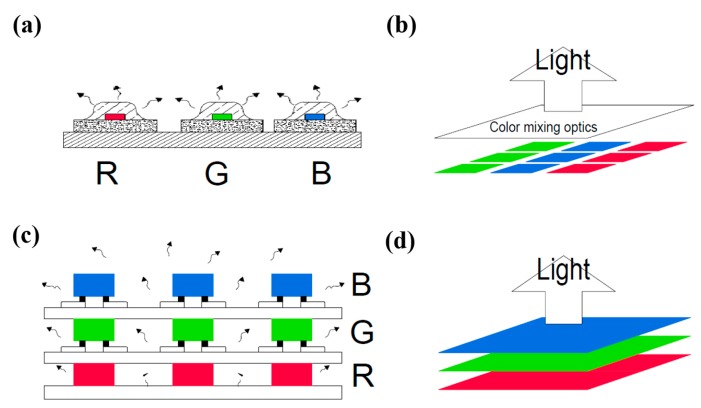
(**a**) The commercialized structure of an RGB tunable LED structure; (**b**) 3D view of the array-type package LEDs; (**c**) the proposed stacked RGB LED modules with transparent glass circuit board (GCB); (**d**) 3D view of the stacked RGB LED.

The commercialized structure of an RGB tunable LED is shown in Figure 1a [12]. A major drawback of this approach is the spatial color variations that give rise to inferior color mixing as emission cones from the discrete devices do not overlap each other completely. In addition, the size of each RGB LED chip is typically small, which also sets limitations on the overall output power that can be delivered for this kind of tunable LED. Although an array-type package can solve the low power output problem, the non-uniform color mixing problem still exists. As shown in Figure 1b, an additional optic lens is used to correct the far field color distributions. Furthermore, different types of packages are adopted to produce color-tunable LED modules [13]. Alternatively, flip chip (FC) technology is widely used [12,13,14,15,16]. However, most FCLED package submounts used today, whether PCB or MCPCB, are still not transparent, which theoretically decreases or even prevents the output light extraction. In view of such limitations, a stacked LED structure with a transparent submount is proposed and shown in Figure 1c,d, whereby multiple RGB LED chips are placed on a transparent submount first and then physically stacked on top of each other [15]. The light paths of the three layers become aligned with each other, producing a beam of light emission that is naturally mixed without additional optics [14,15,17]. A major challenge with this design is that the submount must be transparent for each layer, which the RGB LED chips can be bonded onto them and the light emitted can easily pass through. That is based on one of our previous works in 2015 which adopted transparent submounts for the FCLED and resulted in good light extraction properties [14]. 

For the comparison of lighting properties between the 2D and 3D LED structure, Cheung et al. had built up a stacked (3D) color-tunable LED device and then compared with commercial RGB LED (2D). After their simulation and test validation, the 3D LED exhibits an overall homogeneous emission and reveals a visual proof of satisfactory internal color mixing [15]. Thus, in this study, we propose a stacking multi-chip RGB LED module with a transparent submount which exhibits homogeneous light mixing. Furthermore, the grown Green or Blue InGaN LEDs are also transparent, which benefits light penetration or passing through the proposed stacking module [15].

In reality, despite improvements in the color homogeneity, increasing cost and heat accumulation around the proposed RGB stack LEDs remain severe. It is apparent that in order to solve the problem, a low-cost transparent submount in a stacked LED module must be chosen. In addition, the indium tin oxide (ITO) wiring coated on glass substrate is an effective way to reduce the reflection [18,19]. Furthermore, when multi-chips stack together in a high-power operation, the parasitic heat accumulation must also be solved simultaneously because one major factor in determining the lumen output of an LED is its junction temperature. As junction temperature increases, the light-generation process becomes less efficient and fewer lumens are emitted. For this reason, LED chips generally require a good thermal handling system. However, even in a designed product, the junction temperature may rise significantly above zero bias conditions. Thus, in this paper, an RGB transparent and cost-effective glass stacking structure is demonstrated together with oil encapsulation, which helps to overcome the problems described above.

Nowadays, the phenomenon of ‘efficiency droop’, which comes from a reduction in external quantum efficiency (EQE) since the GaInN-based blue LED operates at a high injection current, has attracted numerous scientists’ attention in the past decade [20,21,22,23]. Frustratingly, to directly overcome it is still difficult due to many physical mechanisms—such as the band-bending of the electron leakage [24,25], carrier overflow from the active region [26,27], and Auger recombination [28,29,30]—have been suggested. Here we selected the aspect of material, which means the cooling oil filling into the LED module, to indirectly reduce part of the efficiency droop from our proposed OCP LED modules.

Of course, there is also the important consideration of color quality that likely affects the LED efficiency. At a specific color, temperature requires a certain spectral content of a light source. Therefore, LEDs with different values for color temperature (CT) or color rendering index (CRI) have different efficiencies. Higher CRI requirements are more restrictive of spectral content and in general tend to be less efficient. In addition, for any kind type of LED, the use of lenses, reflectors, or other special structures to change a product’s spectral distribution also affects the amount of light emission.

To detail the heat accumulation phenomenon in our proposed stacking GCB FCLED module, the power- or current-dependent electrical and optical performance was measured for both single device and multi-chip array, for different colors. TracePro software (TracePro 7.0, Lambda Research Corp., Littleton, MA, USA) was used to check the corresponding optical mounting design.

## 2. Simulation and Experiment

We have observed that the proposed transparent mounting LEDs yield high output luminous flux compared to the traditional mounting LEDs through the simulation results in our previous work [14]. This is due to the fact that total number of emitting rays is larger for the transparent mounting system, which has no rays blocked by the nontransparent traditional submount. Thus, in this study, transparent module material–glass—was selected due to its low cost, high yield, and high transmittance. Thus, the red, green, and blue (RGB) LEDs were sequentially stacked on top of the glass module using the flip-chip technology in a vertical manner to achieve homogeneous light mixing and color-tunable lighting. The red (630 nm, GaAs-based), green (540 nm, GaN-based), and blue (455 nm, GaN-based) LED chips were purchased from Epistar Corp., Hsinchu, Taiwan. The sizes of these chips are 1 × 1 mm^2^.

The first step in the device preparation was to clean the glass submount using acetone and IPA to remove unwanted contaminants. These electrodes were fabricated using photo lithography technology and the E-gun deposition system to deposit connecting patterns on top of different glass modules. Although it is easy to use ITO as transparent conductors in the industry, in this study, a thin narrow visible metal wire was exhibited due to easy pickup and placement of LED dice to the desired pads. It is not difficult in the future ITO wiring at real industry applications. Since the circuit was deposited on glass, therefore we named it the glass circuit board (GCB). Next, the RGB LED chips and fabricated modules were bonded together to form modules for each RGB color or other mixing colors, respectively. 

Before the triple RGB modules were assembled, individual R, G, and B LEDs were immersed in heat sink oil in a color comparison tube to study their output enhancement behavior. A UV LED was also used in the study as a reference. Two kinds of oil, mechanical lubricating oil and transformer oil, were chosen by their own features, such as high thermal conductivity and specific heat, low viscosity, low freezing point, high flash point, low corrosivity, and thermal stability. In this case, the selected transformer oil (MICTRANS-G, Michang Oil Ind. Co., Ltd., Busan, Korea) can keep its own performance under 200 °C due to its flash point of 308 °C, and the highest operating temperature of the LED chip is less than 61.2 °C (@150 mA). In the Appendix A, there is a table shows the product information bulletin of the transformer oil from the manufacturer. Hence, the possibility of photodegradation in the transformer oil is almost negligible in our experiments. After comparing their transparency in the visible light region, transformer oil with higher transmittance was adopted in this study to compare with the lower lubricating one. 

**Figure 2:**
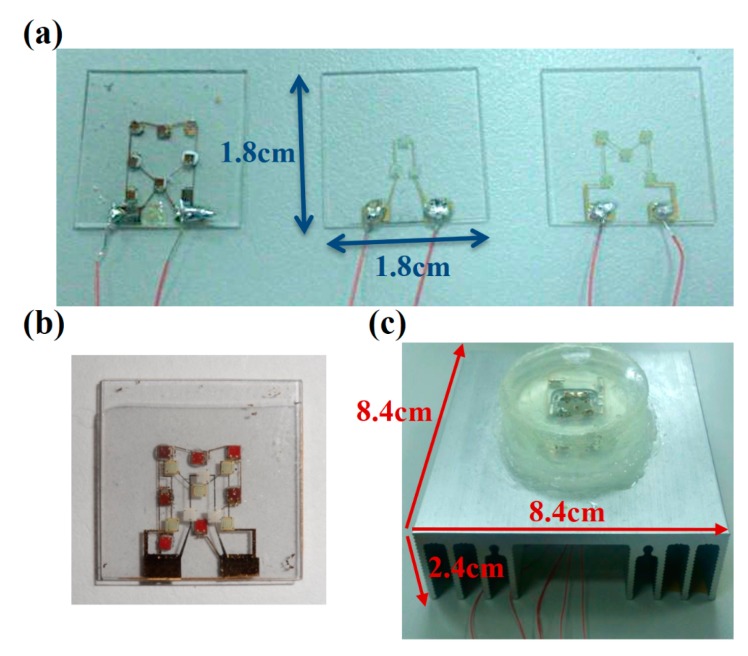
The fabricated stacked RGB LED module. (**a**) The red, blue, and green LED arrays and their connection wires were mounted on the individual GCB; (**b**) top view of the array arrangement of the stacked RGB LED; (**c**) the stacked RGB LED module with cooling oil and aluminum heat sink.

After all the multi-chip LEDs were assembled and FCs mounted on their corresponding GCBs, the red LED module was first stacked inside a quartz cylinder container on the top of an aluminum heat sink, followed by the green LED module, and finally the blue LED module on the top. Small aluminum bumps were intentionally made on the corners of each module to leave a small gap in between, which allowed cooling oil reach them and dissipate heat using the convection mechanism. Finally, the whole set was filled with heat sink oil (transformer oil) and covered with a quartz cap to prevent the cooling oil from leaking. Cooling oil was poured into the device to fill all the inside space. Silicone gel was used to seal the quartz cylinder, the aluminum heat sink, and the quartz cover. Finally, stacking was carried out to accomplish the final structure. Different colors could be illuminated from the fabricated multi-chips RGB stacking LED modules by modulating their individual operating currents. Figure 2 displays the fabricated stacked RGB LED modules with GCB. The Ti/Au patterned wires on GCB surface were fabricated by photo lithography and thermal evaporation equipment and then soldered with tin paste to fix the copper wires, as shown in Figure 2a. The size of GCB plate is 18 mm × 18 mm × 4.2 mm. Figure 2b shows the array arrangement of the stacked RGB LED. Figure 2c indicates the LED modules with cooling oil and aluminum heat sink. The thickness of GCB is 0.7 mm and the distance of each GCB is 1 mm. The cooling oil container was fabricated by quartz ring and the bottom optical glass. The sizes of oil container and the heat sink are 44.5 mm (diameter) × 10.7 mm (height) and 84 × 84 × 24.5 mm^3^, respectively.

**Figure 3:**
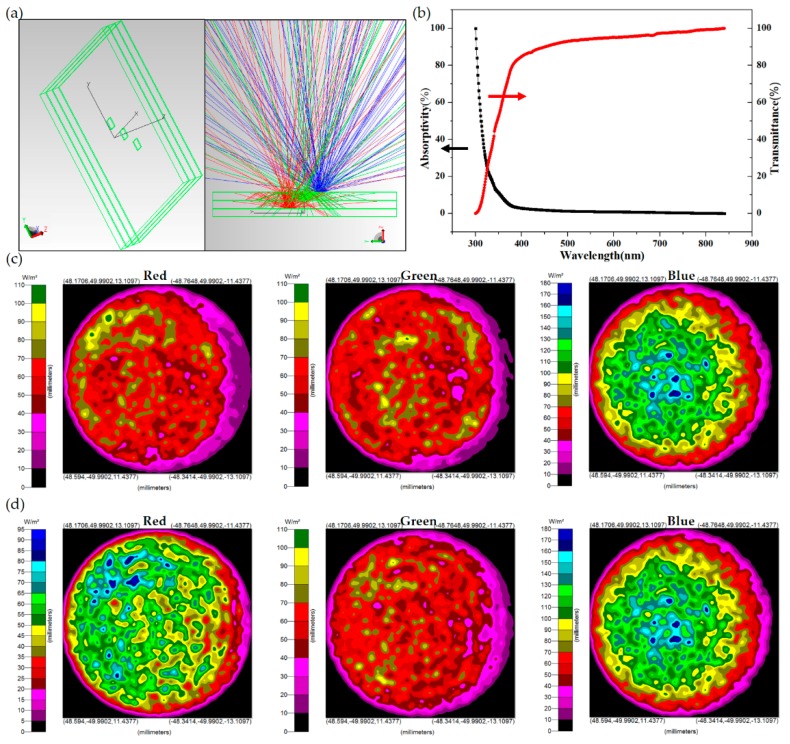
Comparison of luminous flux with different sub-mounts under vertical mounting technologies. (**a**) **Left**: The model of the proposed three GCB layers and FCLED mounting structures in TracePro software; **Right**: TracePro ray tracing results for the RGB LED module with cooling oil; (**b**) the measured transmittance and absorptivity of cooling oil; (**c**,**d**) the irradiance map for absorbed flux of the RGB LED module with and without cooling oil.

Before the output power was measured, as shown in Figure 3a, the model of the proposed three GCB layers and FCLED mounting structures was demonstrated in a TracePro simulation package. The TracePro software was mainly used to simulate the light output efficiency of our proposed FCLEDs mounted on transparent GCBs with or without oil encapsulation (ECP) in an optical domain. The transmittance (T) and reflectance (R) measurements for the transformer oil was carried out using UV–vis/NIR spectrophotometers (V-670, Jasco, Pfungstadt, Germany), and the absorptivity (A) of the transformer oil was calculated by A = 1 − T − R.

For the transformer oil, as shown in Figure 3b, in the short wavelength region (the UV light), the transmittance was low due to the absorptivity. However, for the RGB solid-state lighting applications, the wavelength was longer than 430 nm, which all process a transmittance higher than 0.85. The trance simulation parameters used in this study are listed as follows: each LED emission flux was 1 watt; the angular distribution was Lambertian; the wavelengths were 455, 540, and 630 nm; each submount’s refractive index and absorption coefficient were 1.47 and 0.002895 cm^−1^ at 455 nm, respectively; the cooling oil’s refractive index and absorption coefficient were 1.5 and 0.04831 cm^−1^ at 455 nm, respectively; and a 100-mm-diameter integrating sphere radiation uniform source (ISURS) simulation was described. After simulation, we found that light output stayed more or less at a certain level, although the outside environment of stacked RGB LEDs varied with fulfilled heat sink oil. As shown in Figure 3c,d, the oil encapsulation LED in red, green, and blue rays exhibit 11.3%, 2.8%, and 0% improvement respectively in average emission ray intensity (as shown in Figure 3d) compared to the conventional LED (in air and shown in Figure 3c), which is thought to be due to the graded index effect of the oil (refractive index n = 1.5) between the GaN LED (n = 3.4) and the air (n = 1). The calculated output luminous flux enhancement in air and oil encapsulation is also listed in Table 1. Here we have to emphasize that these simulation or calculation results are based on their optical properties only, no heat accumulation problems are involved or considered.

Later we measured the real light output of GCB modules by using the integrating sphere, which multi-chip RGB LEDs are mounted on each corresponding GCB transparent submounts, respectively. Thus these results were also used to correlate with the experimental heat dissipation results using an infrared (IR) spectrometer.

## 3. Discussion

### 3.1. Single RGB LED with and without Oil ECP

In our previous research, the flip-chip light-emitting diodes were mounted on transparent sapphire and glass, and both have shown a higher output luminous flux when compared to the traditional nontransparent mounted LEDs [14]. Unfortunately, as also shown in that study, the most cost-effective transparent submount GCB is not good thermal conductor, and heat accumulation severely affects its output performance as compared to those FCLEDs mounted on the high thermal conductivity sapphire substrate [15,16,17,31,32]. To solve this problem, an oil ECP technology was proposed in this work [6].

**Figure 4:**
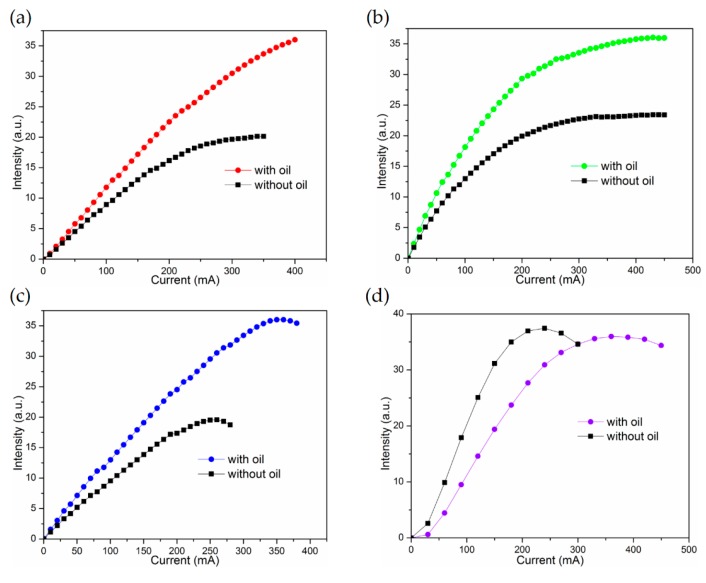
(**a**) red LED; (**b**) green LED; (**c**) blue and (**d**) UV LED L-I curves for the single LED dies, with or without heat sinking oil, respectively.

Before fabricating the stacked RGB LED modules, each single RGB LED’s output intensity versus bias current (L-I) characteristic was measured at first. These single RGB LEDs were all soldered with two tiny bias wires and put into in a color comparison tube, both with and without oil. Their L-I curves were taken and are shown in Figure 4a–d. There are two trends that we can focus on: the output intensity saturation or even drooping phenomenon in the samples without oil encapsulation (OCP) and the obvious RGB output intensity enhancement in the samples with OCP. In addition, you can find that the shorter the LED wavelength, the smaller the OCP induced output intensity enhancement at a certain bias current, such as 80 mA. That is thought to be due to the absorptance phenomenon of the oil. In the meantime, as shown in Figure 4d, in an extreme case, a large UV light output intensity decrease for the OCP UVLED was obtained, which is totally different from its long wavelength OCP FCLED counterparts’ behavior. That is, the UV LED’s output intensity was degraded by the OCP and consistent with the UV–vis measured data which inhibited a large absorptance in the UV region, as shown in Figure 3b.

### 3.2. GCB FCLED Stacked Module with and without Oil ECP

**Figure 5:**
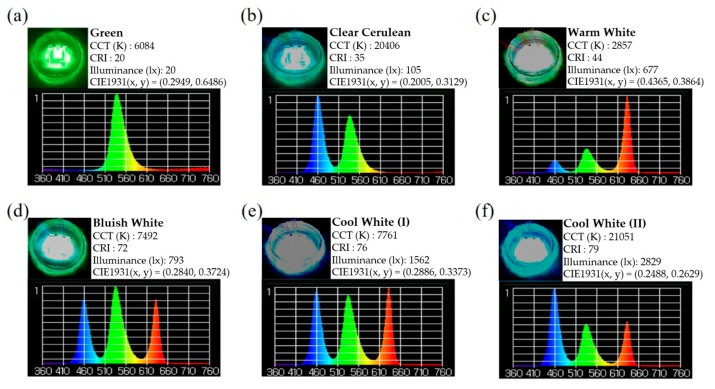
(**a**–**f**) The actual color lighting and its distribution curve of the proposed vertically stacked RGB LED module by different individual operation currents. (CCT: Correlated color temperature) The measurements were made with spectrometer (UPRtek MK350, Miaoli, Taiwan).

Before the requirement of the 3-D package controllable electrically, each layer of the proposed RGB stacked devices was connected individually. That gave individual anodes, while interconnecting their cathodes, resulting in a four-terminal device. When all three devices were illuminated, the mixed light output resulted in different chromatic light, or white light with the right proportions of RGB LEDs. There is also a simple logic behind the vertical stacking structure mounted on the glass submount; that is, the higher energy gap module (such as the blue LED module) should stack on the lower one (the green LED module), and the lowest energy gap module (the red LED module) is kept at the bottom. As a whole, the bottom module is the red LED, followed by the green and then blue LEDs accordingly, as shown in Figure 1c. They are all mounted together through the transparent GCB to produce a naturally uniform mix of light without any extra optic lenses, as shown in Figure 5a–f.

Next, the red, green, and blue light LED modules were mixed at different operating current amounts in order to emit white light with color temperatures between 3000 K and 8000 K. When we adjust the operating current of red, green, and blue LEDs from 30, 20, and 5 mA to 20, 120, and 30 mA, the CT is changed from 2932 K warm, white light to 7998 K cold, white light, which has the highest color rendering index (CRI) of 78. Of course, the CRI can be further improved by changing the RGB LED chips on each module to a proper number [12]. Based on the light distribution curve, as shown in Figure 4, our proposed RGB vertically stacked LED module has a good light output intensity and homogeneous distribution at different angles.

**Figure 6:**
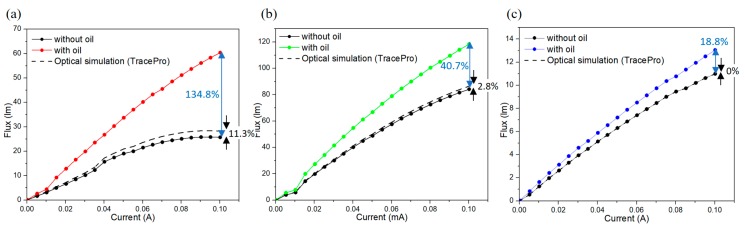
(**a**) Red LED; (**b**) green LED; and (**c**) blue LED L-I curves for the fabricated RGB LED modules.

The measured output intensity behaviors of our proposed stacked GCB LEDs with OCP are shown in Figure 6a–c. Because the light path for each RGB layer with GCB is different and much more complex than the individual RGB LEDs discussed in the section above. Thus, the simulation was carried out in advance and has shown that theoretically OCP does make a small difference (11.3%, 2.8%, and 0% enhancement in the red, green, and blue LED, respectively) in the light output intensity, as shown in Figure 3c,d and Table 1.

For the GCB RGB FCLED modules, before being encapsulated with oil, their output lumens increased with the input current at first, started to saturate, and then decreased at an input current larger than the value of 300 mA, which is well known as the light output intensity thermal droop phenomenon. In our case, all RGB FCLEDs mounted on low thermal conductivity GCB submounts made this phenomenon more significant. After the whole container was encapsulated with the transparent oil, the droop situation was solved immediately. As shown in Figure 6a–c, we also found that the degree of the output intensity enhancement in the RGB modules was different from the single RGB LEDs. As shown in Figure 4a–d, because each single RGB LED was put into the middle of each color comparison tube and their measurement environment was identical. In this stacked GCB structure, the bottom red GCB module was placed on top of a heat sink, it possessed contact conduction and oil heat convection. On the other hand, in green and blue modules, OCP can be more critical. As a whole, the GCB RGB modules with OCP all exhibited obvious increases in their output intensity and can be extend to the higher operating current without saturating or the drooping phenomenon.

It is generally believed that operating temperature is the significant environmental factor that affects the external quantum efficiency (EQE) in LED. In Figure 6a–c, the cooling oil improves GCB LED modules’ light extraction efficiency. The optical simulation in Figure 6 (dotted line) were calculated by multiplying the enhancement rate (as shown in Table 1) from the no-oil measurements of the fabricated RGB LED modules. In these measurements, even if the effect of theoretical optical enhancement is deducted, a 134.8% increase of the red OCP LED compared to the ones without oil at an operating current of 100 mA. The same situation occurs in green OCP LED with an increase of 40.7% at the same operating current of 100 mA. In the case of blue OCP LEDs, the efficiency enhancement is 18.8% at an operating current of 100 mA. All these results demonstrate that the GCB stacked LED modules with OCP is a reliable method to vertically stack multiple RGB LEDs together which can increase the EQE of the correspondent RGB LEDs by removing their heat accumulation effectively. 

**Figure 7:**
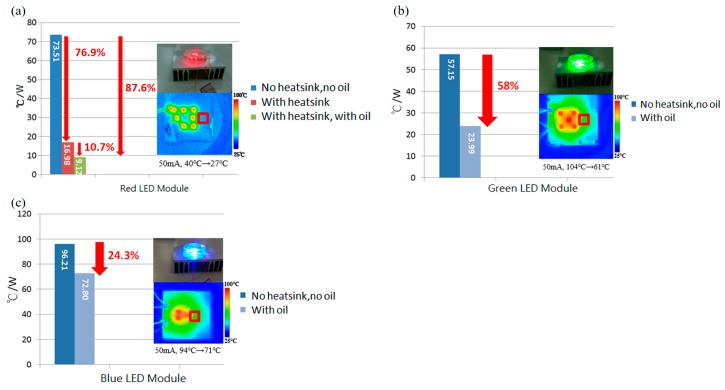
(**a**–**c**) The comparison of heat resistance of red, green and blue FC LED module on the correspondent GCB.

To further detail the correspondent heat accumulation problems, we also applied an IR image system (SC620 Infrared Camera, FLIR^®^ Systems, Inc., Wilsonville, OR, USA) to obtain the temperature value of the respective GCB LED modules; their corresponding heat resistances were calculated and are shown in Figure 7a–c. The heat resistance of the red light module was 73.51 °C/W before coolant oil packaging and 9.12 °C/W after packaging, for a decreased rate of 87.6%, as shown in Figure 7a. Of course, for the bottom red module, only accounting for the oil convection effect, a 46% decrease (16.98 °C /W → 9.12 °C/W) was obtained. The heat resistance of the green light LEDs’ module, the middle layer, was 57.15 °C/W before packaging and 23.99 °C/W after packaging, for a decrease of 58%, as shown in Figure 7b. The blue light LEDs’ module in the top layer, which is far from the heat sink, was 96.21 °C/W before packaging and 72.80 °C/W after packaging, for a decrease of only 24.3%, as shown in Figure 7c. Thus, as shown in Figure 7a–c, the red module exhibited both heat conduction and heat convection, which made it the best heat dissipation performer; the middle green and top blue modules exhibited weak conduction and different degrees of heat convection, making them have different heat dissipation improvements with the proposed OCP cooling performances.

## 4. Conclusions

Based on our RGB vertically stacked LEDs’ module on transparent GCB, various color mixing can be easily accomplished as a power color-tunable device with a wide range of naturally mixing colors. It is shown that all the different color output lumen of our proposed RGB LED, multi-chip modules, packaged with transparent GCB and heat sink OCP, their corresponding lumen output, showed obvious enhancements such as 134.8%, 40.7%, and 18.8% respectively. A consist thermal calculation of GCB LEDs with OCP is also obtained, the heat resistance of the bottom red light LED module decreased by 87.6%, the middle green light GCB LED module decreased by 58%, and the blue light GCB LED top module decreased by 24.3%. In addition, in the GCB LED modules, the heat conduction of the red module and heat convection of the RGB modules have better heat dissipation improvements, especially in the elimination of the high-power output saturating phenomenon.

Nowadays, the commercial low and middle power LED devices are well established and the lighting industries tend to study the high output power ambient light application. Once the heat problem of GCB can be further mitigated, we believe there will be many possibilities for transparent GCB in the field of high output color tunable LED modules in the future due to its high light extraction ratio and nature color mixing when compared with the present nontransparent MCPCB mounting method. 

## Figures and Tables

**Table 1:** The theoretical output luminous flux enhancement in oil encapsulation.

LED Type	Normal (in Air)	Oil Encapsulation
Red	0.6235 W	0.69378 W (11.3%↗)
Green	0.66853 W	0.68732 W (2.8%↗)
Blue	0.99898 W	0.99898 W (0%↗)

## Appendix A

**Table A1:** The product information bulletin of the transformer oil.

Property		ASTM Test Method	MICTRANS G
**Color**	ASTM	D1500	L0.5
**Flash Point**	°C	D92	308
**Interfacial tension**	25 °C, g/cm^2^	D971	41.5
**Pour Point**	°C	D97	−33
**Specific gravity**	15/15 °C	D1298	0.8697
**Viscosity cSt**	100 °C	D445	13.7
	40 °C		126
	0 °C		2223
**Neutralization number**	mgKOH/g	D974	0.007
